# The voices of incarcerated women at the forefront of parenting program development: a trauma-informed approach to education

**DOI:** 10.1186/s40352-022-00185-7

**Published:** 2022-07-13

**Authors:** Belinda J. Lovell, Mary P. Steen, Angela E. Brown, Adrian J. Esterman

**Affiliations:** Clinical and Health Sciences, University of South Australia, Adelaide, Australia

**Keywords:** Women, Mothers, Incarceration, Parenting, Education

## Abstract

**Background:**

The lives of women experiencing incarceration are complex, impacting many aspects of parenting. Incarceration can present an opportunity for women to access parenting education. However, their specific needs have to be considered. Few parenting programs for women experiencing incarceration have involved the women as part of their development.

**Methods:**

Six focus groups were conducted in a prison setting involving thirty-one women to explore and understand their parenting education needs.

**Results:**

Four main themes were identified to reflect the complex lives of the women and their parenting education needs. These themes were: working towards a positive self, communication as a lifeline, supporting and nurturing their children and hopefulness and reconnecting. The trauma women experienced in their lives was apparent during discussions.

**Conclusion:**

Women requested a non-judgmental parenting program to be developed to meet their specific needs and circumstances. The program needed to be designed to enable them to share stories with women in similar situations. Women gave insights into some of the specific content and topics they would like included in a parenting program. The women revealed experiences of trauma in their lives, demonstrating the importance of the need for a trauma informed approach to parenting education.

## Background

The majority of women experiencing incarceration have complex histories which can include child abuse, sexual abuse, neglect, domestic violence and drug and alcohol misuse (Bartels et al., 2020; Segrave & Carlton, 2010). These life experiences can impact a woman’s capacity to maintain employment, impact her mental health and create issues with parenting (Stathopoulos, 2012). Parents who have experienced poor parenting in their childhood are more likely to continue poor parenting with their own children (Kim, 2009). Many women in prison are mothers or in mothering roles and in Australia 85% of incarcerated women report being pregnant and the majority of women have at least one dependent child (AIHW, 2020 pp. 10). The increasing population of women in prison has left many children separated from their primary caregiver and thus mothering whilst incarcerated has become a serious issue for these women and hence the need for parenting education (Stone et al., 2017; Tuerk, 2007; Walmsley, 2016). In Australia, the over representation of Aboriginal and/or Torres Strait Islander^1^ peoples in prison needs consideration. The rate of Aboriginal women experiencing incarceration in Australia is 35 times higher than all other women in Australia and equates to 34% of the female prison population, and the majority are mothers (AIHW, 2019 pp. 72). This over-representation has been attributed to the devastating impact of the British invasion of Australia, and the forced removal of children by the government, referred to as “the Stolen Generation” (AIHW, 2018; ALRC, 2018).

Imprisonment can present as an opportunity to attend parenting education that may not have been accessible in the community (Fowler et al., 2018; Miller et al., 2014). Nevertheless, it is currently difficult to recommend the structure and content of education required for a parenting program for incarcerated women, as previous programs have varied in approach and content. While no “gold standard” program can meet all the needs of incarcerated women, well-researched programs are needed with defined participants, methods, and goals (Loper & Tuerk, 2006). Prison parenting programs have to date mostly focused on parenting from prison (Kennon et al., 2009; Urban, & Burton, B., 2015); parenting and the child relationship (Perry et al., 2009); parenting, relationships and reunification (Wilson et al., 2010); parenting skills, behaviour and relationships (Miller et al., 2014); parenting and relationships using Cognitive Behavioural Therapy (Collica-Cox, 2018; Loper & Tuerk, 2011; Simmons et al., 2013) and parenting and relationships using Attachment Theory (Bell & Cornwell, 2015; Kamptner et al., 2017). Prison parenting programs include topics such as, improving relationships with the child, caregiver and family; communication and listening; taking responsibility for the crime; child discipline; emotional reactions of the parent; child development; effective parenting skills; managing post release and self-esteem (Lovell et al., 2020a). To inform future development of programs, common problems have been identified from previous program evaluations - for example, lack of child contact to enable consolidation and practice of parenting skills (Loper & Tuerk, 2006; Perry et al., 2009) and program attrition (Lovell et al., 2020a). Programs reporting significant positive impacts have required intensive time and resources, and ideally continue after prison release in a community setting (Frye & Dawe, 2008; Menting et al., 2014; Shortt et al., 2014). Additionally, programs need to address low levels of literacy (Miller et al., 2014), provide support for women if they experience distress, and have more than one facilitator (Perry et al., 2009).

Armstrong et al., (2018) in a systematic review and meta-analysis reported a short-term increased moderate effect when measuring the quality of parent–child relationships following attendance at parenting programs in prisons. The authors agree with previous reviews, that parenting interventions can be adapted for prison settings and have a positive impact on parental and family wellbeing. Due to the complexities of incarcerated parents’ lives, they recommend programs include parenting, as well as other needs such as substance abuse, and include a trauma-informed approach (Armstrong et al., 2018).

In the past the majority of prison parenting programs have not considered the cultural and contextual needs of participants (Brown & Bloom, 2009; Henson, 2020). Examples of the contextual needs, include separation of mother and child, distance of the family from the prison and the unique needs of parents in the prison environment (Henson, 2020). The literature suggests that to improve positive outcomes, programs must consider the needs of women and are reflective of the prison environment (Aiello, 2016; Loper & Tuerk, 2006; Troy et al., 2018). Parenting from prison has unique challenges for women, often associated with increased anxiety and stress (Loper et al., 2009). Prison-specific considerations that may need to be addressed have been highlighted by Loper and Tuerk, (Loper & Tuerk, 2006) such as the varying length of sentences women have to serve, the amount of child contact, prior history, relevant topics, age of the children, cultural background, literacy level, and security restrictions.

To accommodate the specific needs of women, a gendered approach to programming needs to focus on relationships, strengths of the women, their cultures and be sensitive to trauma (Bartels et al., 2020). A trauma-informed approach promotes a positive outlook i.e., that relationships can be reconciled, and recovery is possible (Kezelman & Stavropoulos, 2020). This strengths-based approach has been reported by Kezelman and Stavropoulos (Kezelman & Stavropoulos, 2020) to instil hope and optimism and empower women to feel that recovery is possible. It is guided by six core principles: safety; trustworthiness; peer support; empowerment, voice and choice; collaboration, and cultural, historical and gender issues (SAMHSA, 2014). To accommodate the needs of incarcerated women and children, the United Nations General Assembly (2011) developed ‘The Bangkok Rules’. These rules established standards for incarcerated women globally and include a recommendation that programs, including parenting programs, are designed specifically to meet the gendered needs of women. To fulfill the Bangkok Rules, women experiencing incarceration need to be given a voice to understand their parenting education needs. Currently few studies have directly discussed parenting education needs with the woman who would participate in a program. The voices of incarcerated women have often been ignored and it has been recommended that more research is needed that uses the voices of incarcerated women in program development (Kennedy, & A. M., & Allen, C., 2020).

### Needs assessment

Linhorst (2002) has highlighted that a needs assessment for planning programs can be conducted by using focus groups and is an effective way of collecting information and can be used for sensitive topics. Focus groups have been demonstrated to enhance analytic rigor of previous studies in a prison setting (Eddy et al., 2008; Pollack, 2003). For example, focus groups have been utilized to conduct a needs assessment prior to implementation of prison parenting programs for fathers, which enabled the understanding of their needs and helped to address the most important issues identified by male prisoners (Henson, 2020). The current study used focus groups to explore and understand the needs of women experiencing incarceration, using an exploratory qualitative approach. Focus groups reported in this paper are part of a larger research study using a mixed-method, multi-phase design, framed by a community-based theoretical model which promotes collaboration between researchers and community to address a community issue (Lovell et al., 2020b). This study design and methodology is valuable for understanding populations that have been marginalized, giving them a voice and empowering them (Badiee et al., 2012). The purpose of the focus groups was to explore the parenting education needs of women experiencing incarceration in South Australia to co-create a parenting program that would address the specific needs of these women.

## Methods

### Study design

To understand the parenting education needs of women experiencing incarceration, data were generated during six focus groups undertaken in a designated room in a prison setting in South Australia. The prison guards were outside the room during the focus groups, which provided some privacy and confidentiality. The women were assured they could leave at any time or not participate if they decided not to contribute. Six focus groups involving 31 participants stimulated in-depth discussion to gain a deeper understanding of the needs of incarcerated women and facilitate discovery of new insights (Breen, 2006). The women discussed topics to include in a parenting education program, what outcomes they wanted, the type of education and helpful activities. The women discussed issues they identified as incarcerated mothers and parenting support that would be useful. Examples of some of the questions asked during the focus groups included:

What are some of the topics you would like to learn about during a parenting education program?

If you did attend a parenting program, what would you aim to gain from attending?

What type of education would you find helpful? i.e. group, individual.

What do you see as some of the problems or issues that you have as a mother?

### Eligibility

Women were eligible if they were able to speak English, as English is the primary language of 90% of Australian prisoners (Australian Institute of Health and Welfare [AIHW], 2019, p. 13). They were also required to understand the study information and identify as a mother or carer for children of any age (including adult children) residing in the community. Women were not required to have contact with their children to be involved. The prison site where the study took place did not accommodate children living onsite.

### Recruitment process

Eligible women were given a brochure about the study by two Correctional Activity Coordinators in the prison and invited to attend an information session. A combination of convenience and purposive sampling was used. To represent population diversity, women who identified as Aboriginal and/or Torres Strait Islander or were born overseas were specifically asked if they would like to attend an information session. During the information sessions the women were informed of a specific focus group for Aboriginal women and migrant women and were able to decide to attend the specific session, or a group open to any eligible women. The primary researcher led information sessions with the support of MS or AB. Information sessions involved discussions about the study, participation requirements, and the voluntary nature of the study. All women were able to communicate in English. Women were recruited during these sessions by researchers who had no prior relationship with the women. Six information sessions were conducted and consisted of less than ten women, to enable researchers to communicate directly and clearly with each woman. All information about the study including consent forms and participant information sheets were given to women and read aloud by researchers. Time was spent during information sessions to ensure questions were answered. A simple overview of the main points was reiterated, and it was highlighted that women should not communicate any illegal activity they had been involved in and discussions should remain confidential. Women were made aware that any disclosures could not be guaranteed to remain confidential and therefore should only share information they were comfortable sharing. Women were informed that the information obtained through the focus groups would be used to develop a parenting education program. The women were reassured their decision to participate or not, would not impact any aspect of their incarceration. Nine out of 40 women who attended an information session decided not to participate in the study. Six women did not give a reason. However, some reasons reported for not participating included pending court appearance, wanting to talk with an Aboriginal Elder but not participate, not wanting to take the potential risk of information being shared for legal purposes.

### Focus groups

The six focus groups were conducted in the women’s prison over five months (December to April 2020). During the focus groups, discussions were commenced after gaining written consent from each woman. The focus groups included a mix of women on remand (yet to be sentenced), and some with short and long sentences. Focus groups were facilitated by female researchers, the primary researcher BL (PhD candidate), co-facilitated by MS (Professor) and one by AB (post-doctoral early career researcher). The researchers identify as white, middle-class academics with strong interests in health equity and advocate for women and babies within policy, research and their profession. The authors see themselves as outsiders to the prison environment and brought to the research knowledge from their experiences and engagement with vulnerable women in the health care setting, understanding of the literature around women in prison, previous evaluations of prison parenting programs, understanding of the impact of colonization on Aboriginal peoples and the principles of trauma informed approaches.

### Participants

Two focus groups were undertaken in high security and four in low security sections of the prison. To represent population diversity Aboriginal women and women born overseas attended two separate focus groups and some attended general focus groups. A female Aboriginal Elder was present during the focus group offered to the Aboriginal women to provide support. Between three and eight women attended each group, with an average of five women per group. Women were aged between 19 and 60 with an average age of 35 years. Twenty-six women were born in Australia, two in the United Kingdom, one from New Zealand, Southeast Asia, and Northern Europe. Of the women born in Australia, thirteen identified as Aboriginal. Two women were not biological mothers and cared for children of relatives. Other women had between one and five biological children, ranging in age from newborn to adult children in their 30’s, and a number of women were grandmothers. Between the women there were approximately 70 children. Seventeen women recorded their sentence length which ranged from six weeks to more than 25 years, with an average of five years. Eleven women were on remand. Women had varying contact with their children – some were looked after by family, a few reported children in foster care, and many had Child Protection involvement. Child Protection is a South Australian government agency designed to protect the safety of children who are at risk of being abused, neglected, or harmed or their parents are unable to safely care for them (AIHW, 2021).

### Data collection

A naturalist approach enabled us to examine the lived experience of the women with the understanding that phenomenon should be studied in context, giving voice to a marginalized population as described by Given (Given, 2008). A prominent feature of naturalistic inquiry is the understanding that individuals are experts in their own experiences (Ravitch & Carl, 2018). Focus groups were recorded using a digital recorder and handwritten notes were documented on a flipchart by the co-facilitator. Discussing some group communication guidance at the commencement of each focus group helped facilitate conversations and instilled respect to listen to everyone participating. Women were prompted with semi-structured questions by the primary researcher. The women considered questions with one another, stimulating further discussion. This collective and engaging approach was maintained by encouraging stories to unfold without any interruption and analyzing the relationship with the research question after discussion as recommend by Bessarab and Ng’andu (Bessarab & Ng’andu, 2010). The focus group sessions lasted 60–90 minutes. Questions were in a sequence that helped to build rapport and trust and gradually related to more sensitive topics. Questions focused on parenting education and what would be useful in a parenting education program for women during incarceration. Questions were asked about program length, structure, content and activities. Group dynamic issues were managed positively by having two researchers present. Occasionally, some women were particularly talkative and needed re-direction and other women gave minimal verbal input and were prompted to contribute. When the discussion came to a natural end, prompts were used to broaden topics being considered. At the end of each focus group, the co-facilitator verbally summarized the main ideas/topics written on flipcharts. Women were given an opportunity to confirm the ideas/topics and/or dispute any inaccuracies or offer any further suggestions. The researchers present during interviews also conducted the data analysis, strengthening this process. After the focus groups the two researchers debriefed by discussing group highlights, challenges experienced, observations and main themes communicated. Transcription was undertaken directly after each focus group to begin analysis. Researchers agreed that six focus groups had achieved rich data extracts to gain an in-depth understanding of the educational needs of the women (Braun & Clarke, 2021a). A focus group was planned for pregnant women who were incarcerated; however, they could not be interviewed due to the COVID-19 pandemic restrictions.

### Data analysis

The data were analyzed using a reflexive thematic analysis approach (Braun & Clarke, 2019). Six phases of analysis were used to guide the process, as recommended by Braun and Clarke (Braun & Clarke, 2006; Braun & Clarke, 2014). This approach was chosen for its flexibility, researcher subjectivity and engaged approach to theme generation (Braun & Clarke, 2021b). The first phase of immersion in the data commenced whilst conducting focus groups. Familiariziation with the data continued with the primary researcher transcribing the focus group discussions verbatim from digital recordings, removing any identifying characteristics. During this process recordings were listened to two to three times in entirety and transcripts were read and re-read to be immersed in the data and reflect on the data. During phase two coding was conducted by the primary researcher and included both semantic (practical parenting) and latent codes (trauma, connection) using a relativist perspective. In line with Naturalistic inquiry an inductive approach was used to focus on meaning within the data which involved reading and re-reading transcripts (Armstrong, 2010). Codes were highlighted in the transcripts to identify interesting aspects that answered the research question, drawing on the researchers’ values and knowledge. Codes were revised, refined, and collated into a table. This phase was conducted in conjunction with experienced senior researchers and all researchers agreed on the interpretation of data. Themes were generated in phase three by grouping together codes with similar meanings. Women’s individual quotes were assigned to substantiate themes. The primary researcher reviewed transcripts during phase four to ensure overall findings of the data had been represented. Transcripts were uploaded into software program NVivo 12.6.0 to assist with handling large amounts of qualitative data. Themes were reviewed and rearranged to facilitate flow of the overall findings. During phase five researchers discussed themes, named and re-named and defined themes to ensure women’s views and experiences had been interpreted accurately. The primary researcher wrote a reflective journal throughout the process to record thoughts, feelings, and assumptions about the experience of the focus groups and analysis. Further reflection was facilitated from discussion of theme generation with all researchers. The researchers’ depth of engagement with data and the reflexive process was used as the measure of quality practice. This practice differs from other qualitative data analysis frameworks guided by inter-coder agreement (Braun & Clarke, 2021b).

## Results

Initially in the focus groups the women discussed the structure of a parenting program. This discussion occurred to develop rapport and trust with the participants which enabled researchers to then ask questions about more sensitive topics. The discussion progressed to women’s specific needs, to be addressed within a parenting education program.

### Structure of parenting program

The women were positive about the concept of a parenting program designed for their needs. Collectively, women agreed that all child developmental ages should be included: an example, “Need to cover from a young age to an older age because a lot of girls around here either have older kids or really young kids or grandkids that are young as well” (FG 1). One woman suggested it would be a good idea to encourage women to voice their reasons for attending the program to enable the program facilitator to: “Alter it to that person for what they need” (FG 2). Women wanted to attend parenting education once or twice a week, for five to eight sessions, lasting one to two hours. Many women were serving short sentences and for that reason women thought sessions should be offered twice a week. The women requested sessions to be kept short as they recognized some may become restless, bored, and unable to concentrate for long periods. The women described wanting a combination of discussion, videos, handouts, lots of information and some suggested roleplays would be useful. The provision of handouts was recommended to enable women to revisit information and may assist women who have learning challenges as one woman shared: “I have a really bad memory and short attention span” (FG 1).

Most women liked the idea of making and sending something to their children and suggested making a puppet or card with their photograph. The women explained they wanted their children to have something to keep and show that they were okay. A few women proposed that a “lived experience mentor” would make the program worthwhile. This mentor would be a person who had experienced similar circumstance and could communicate about their transition to a positive lifestyle. Interestingly, several women suggested the benefit of incorporating humor into the program: “A funny starter would be good; that will get the girls connected” (FG 1). Many women stated they did not want to feel judged: “There’s gotta be some sort of respect on each level – no discrimination, no judgment” (FG 5). The women liked the idea of peer support, knowing others had similar circumstances. Women expressed how they wanted a facilitator to connect with, to listen, and who would understand and not judge them. The majority of Aboriginal women expressed a need for an education group specifically for them. These women suggested inviting their Elders for circle time as a way of building respectful relationships and passing on cultural knowledge. Connection to culture and community is important for Aboriginal peoples, to maintain their social and emotional wellbeing.

### Working towards a positive self

The first need identified by the women themselves was to work towards a positive self. In all focus groups women communicated trauma experienced in their lives including being in prison and being separated from their children and family. This trauma included domestic violence, lack of support, removal of children and using drugs and alcohol as coping strategies. The Aboriginal women described the added trauma of racism, discrimination and intergenerational trauma. The women recognized the need to look after their own health and wellbeing in order to look after their children. Women described their cycles of distress with domestic violence and having limited skills and support to draw on for their emotional health. The threat of deportation was another issue for women who were not Australian citizens. One woman, however, viewed deportation as a way of making a “new start” and escaping the threat of domestic violence, demonstrating her fear of the situation. Another woman exemplified the severity and frequency of domestic violence when she said:

This whole jail is domestic violence, every single woman in here has had some sort of it, every single one, that and yeah and pretty bad cases. (FG 6)

Domestic violence and related trauma were seen as common amongst the women and the women recognized the need to work on their own healing.

Racism and discrimination were discussed by Aboriginal women in relation to the criminal justice system and Child Protection.

How many different stories there is of indigenous women and men, Elders, all genders and yet we still stand here, with our respect and dignity of who we are, as Aboriginal women. (FG 5)

This woman highlighted the added layers of discrimination and trauma that Aboriginal peoples experience and the strength of their people and culture despite the hardship they have endured.

Women were looking for positive self-care strategies as some discussed not being nurtured in their childhood. One woman highlighted the fact that she had not been shown love and was unsure how to show her children love:

Now if you’ve only been shown one way, like if they’ve never been shown love, how you gonna show em that? (FG 5)

A number of women discussed their mental health and referred to “not coping”, being left with their own thoughts or more specifically depression and anxiety. One woman described escaping the pain by withdrawing and not having the skills to deal with difficult emotions:

Yeah, it’s the honest truth – you leave with some sort of depression, anxiety being around big groups. You can’t handle it because you are so used to withdrawing in because that’s the only way you can deal with being inside here. (FG 4)

The trauma and mental health conditions women experience highlight the importance of building a safe and trusting environment within a parenting education group.

Some women felt stressed and saddened when thinking about the separation experienced from their children and family. One woman reflected on the sadness she was experiencing when thinking about her daughter who was removed through Child Protection and expressed how she feels like a stranger:

I have to let that be, as my own child looking at her, not knowing who I am and that’s what I have to let, because that is what’s happened. (FG 5)

Grief and sadness were experienced by some women as they came to realize their role as a mother had diminished. However, women were motivated to learn skills to deal with difficult emotions. Women described experiencing an array of negative emotions including stress, anger, frustration, guilt, grief, loss, regret, remorse and sadness. Women reported managing their emotions by writing in a journal, attending programs focused on positivity, exercise, breathing and meditation. Some felt uncomfortable with breathing exercises as stated by one participant, “they make me feel a bit stupid” (FG 1) and discussed not being able to sit still for breathing and meditation. Many women discussed the need for more skills and tools to draw upon. One woman remarked,

We are just expected to be like warriors and be able to cope with it all, it’s not easy. (FG 2)

Another woman stated,

We need a skill that is quick and easy to turn to because drugs is quick and easy to turn to. (FG 3)

The women reported trauma and stress experienced in their lives and were needing skills and support to make positive changes to their own health and wellbeing. Women wanted to increase their knowledge and be able to navigate support systems and recognized that the temptation to turn to substance misuse would be difficult to overcome. Their children were central to their desire to make positive changes. The focus for women on a positive future and a history of trauma aligns with the need for a strengths-based, trauma informed approach to education.

### Communication – as a lifeline

Communication and connection with children and family was vital for the women. The main method of communication was through telephone calls. Women expressed their difficulty talking to young children over the telephone. Some women discussed how they took this personally, thinking their children did not want to talk to them. Others expressed how they were not up to date with their child’s interests or milestones. Nevertheless, women placed a significant amount of importance on telephone calls because it was their main form of communication.

What I really wanted to learn is how to communicate with my kids when I am incarcerated and being with them, like with phone calls. (FG 6)

Many agreed the availability of a video call would improve conversations, especially with young children who would often use one-word answers as one woman explained:

I go, ‘How was your day?’ She said, ‘Good.’, ‘What did you do?’ ‘Things.’ I go, ‘What did you learn?’ ‘Not much.’ My son says goes ‘I don’t know, mum she never shuts up at home, she does not shut up and it is only when she is on the phone that she doesn’t talk things up’. (FG 6)

This account demonstrates the challenges of talking to young children over the telephone and some of the women reported feeling unsure of how to improve their conversations. Some women did not know that it is common to have difficulty talking to young children on the phone in any circumstances. The importance of connection with family via video calls was highlighted by one woman:

I’m relying on, when I get deported that it will be my lifeline, will be this skype to talk to my son, granddaughter and my friends. It’ll just be that will be my way of communicating – yeah like so I can still see them. (FG 6)

This woman described the limited opportunity she would have to connect with her family as she held hope that video calls would be available for communication. She described the calls with family as her lifeline.

Several women discussed how they wanted support to improve their written and verbal communication. The Aboriginal women also discussed the need for improved communication skills with professional people, for example, lawyers and social workers which could impact decisions around child contact. Difficulties communicating with people in a position of power may reflect the fear and distrust created by Australia’s history of colonization and forced child removal which led to a complete disruption of all aspects of life for Aboriginal peoples.

Some women wrote letters, journals and sent unfinished coloring pictures back and forth to their children until completion. One woman described the importance of writing in a journal and encouraged her child to read her reflections. This was an effective way to express her feelings and connect with her daughter:

I’ve given it to my oldest and they have read [it], so every time I have been in jail while she was a baby and while she was growing up – she’s read it now. (FG 4)

In one group women laughed and joked that their children would write messages using abbreviations on their computer i.e., LOL (laugh out loud). Many participants reported their children rarely responded because they were too focused on technology, which was illustrated by a woman who said,

It’s all on computer cos, I always say to my kids, ‘send me a picture anything, just send me something,’ you know but they don’t do that these days – they don’t know how to. (FG 4)

Even though women joked about their children not writing, it appeared they genuinely wanted to receive a letter. Children not responding was a shared experience by many of the women and some had given up trying. Communication with family and children was considered important to most of the women. However, the women reported communication problems and conveyed they did not have access to support with these issues.

### Supporting and nurturing their children

Women explained feeling helpless whilst incarcerated and unable to provide support and nurture their children. They spoke at length about problems their children faced daily due to their incarceration as well as trauma their children continue to experience. It was acknowledged that children face challenges such as, what to tell their friends, friends not wanting to play with them, media exposure of their mother and the crime, and the shame of having a mother in prison. One woman explained,

Yeah, and it’s easy for me cos I am out of the scene. They are the ones out there dealing with everyone talking, the shame, yeah oh you can’t play with that kid cause that parent did this – sort of thing – you know, it would be hard. (FG1)

Women were concerned their children were being punished for their mistakes and these challenges led them to want to learn how to support their child emotionally and understand their behavior. Many women discussed children getting into fights at school and being bullied. One woman commented that most kids end up moving school because they “copped a lot” as the women discussed their children having to cope with the shame of having a mother incarcerated and other children not wanting to play with them anymore. The women described being powerless as their child’s behavior changed from doing well to getting suspended, as one woman said,

Now my 13-year-old; he’s running amok at school and getting in fights and carrying on cos I’m in here – yeah behavior – yeah that’s another thing to learn about, yeah different behaviors. (FG 4)

Although, several women discussed problematic behaviors, no one mentioned any external supports with which their children were engaged. Some women expressed worry about their children, but they did not feel in a position to provide support. One woman learnt through her relationship with her son, the importance of communicating that her incarceration was not his fault, and she still loved him. This woman was able to reflect on the behavior of her son and gain insight into his experiences and understand why he was angry. This woman discussed the quality times she spent with her son, participating in activities he enjoyed and recognized the impact of incarceration on their relationship when she had been previously released from prison,

My son thought I didn’t love him anymore because I come [sic] here and so he was very angry, you know this was when he was like 11 years old. And he was very angry, and this was a boy that I would take camping and go motor bike riding, but he just didn’t want to. (FG4)

A few women were anxious because they felt a lack of control over what their children were doing, particularly as older children were gaining independence. One woman highlighted the difficulties associated with parenting teenagers from a distance,

I feel like I should be there, she shouldn’t be just winging it by herself. I feel like I’m going to be a grandmother before I get out, yeah and she’s, yeah, fifteen. (FG1)

A few women reported their teenage children were at a vulnerable stage, negotiating life with limited guidance. Women discussed how they felt connected enough to hear their stories but struggled with the best way to support their developmental needs. Many women expressed how they wanted guidance to be able to talk to their teenagers. The separation and powerlessness women experienced also led to feelings of guilt. Women discussed how they missed out on special occasions and everyday activities like sport or supporting their children with homework. One woman explained,

Yeah, definitely you feel guilty that you missed birthdays, Christmases; you feel guilty that you can’t be there for all those moments. (FG 2)

Another woman explained the guilt as “the worst, the worst” and another as “a big thing” (FG 2). The women who discussed guilt, phrased this by saying “you feel guilty,” rather than saying “I feel guilty”, as a way of acknowledging that most women in the prison experienced guilt.

Supporting the children to understand the circumstances as to why they were in prison was a concern for many women. Women discussed the need for guidance to inform their children as to why they were in prison, about the crime committed and how to make the conversation age appropriate. It became apparent women had not been given any specific guidance about communicating their circumstances with their child. One woman explained,

Yeah, it’s not their fault for what we have done but it’s hard to try and explain it to them, that you are sorry – you know ‘cos from their point of view it’s like they are copping it for something that they haven’t even done. And we are trying to say – what can we say, you know. (FG1)

Some women told their children, “Mummy was naughty,” and “they have to stay in jail” or they had not told the child and were thankful they would not remember. Other difficult conversations were discussed, particularly with teenagers such as committing a crime and “hope that they don’t follow mum’s footsteps” (FG 4), teenage pregnancy, and other problems their child may face were cause for concern. Another woman explained a conversation she had with her eighteen-year-old daughter on the telephone, who was missing her mother and threatened to get herself incarcerated to spend time with her:

‘I’m so going to get myself locked up so I can come spend time with ya’. So, I had to get angry with her on the phone; it was the worst feeling ever and ‘you can’t be coming in here’. ‘It’s not that bad in there’ and then when I told her and I had to say: ‘well hang on a minute, what you see is nothing, yeah.’ (FG 1)

This woman was having difficulty communicating that she did want to spend time with her daughter, however she became angry when trying to discuss how serious she was about not wanting her daughter to end up in prison. The women expressed a deep understanding of how their situations have a negative impact on their children and this too distressed them.

### Hopefulness and reconnecting

The majority of women expressed a strong desire to reconnect with their children and family despite complex challenges, particularly with Child Protection and in some instances the caregiver of their child. Women expressed mixed feelings of hope and concern about reconnecting with their children and returning to the community. A specific need for practical parenting skills and information was deemed essential for the transition. Some women knew their role of mother may no longer be achievable and expressed wanting to give their children space to find forgiveness, without further distancing the relationship:

I think even if you are not in your kid’s life, always just being there. You don’t have to be in their lives but being there so whenever they want you or need you, you are there, that’s the thing. (FG 2)

This woman wanted to stay connected with her children and regain their trust by being available when they were in need. She had come to the realization that her day-to-day mothering role was lost. This may have been her way of coping and embracing her role in a positive way.

Although, the issue of child custody stimulated confusion, sadness and frustration for many, the women were searching for ways to reconnect. Women conveyed not knowing how to negotiate custody issues with their family and Child Protection. Discussions around child custody stirred emotions with one woman expressing:

I know I can’t just go in and rip her out of my mum’s arms, I know that it’s not possible but at the end of the day, she is still my daughter. (FG 1)

The desire to regain the mothering role was evident by this mother “wanting to rip the baby out of her own mother’s arms*”* and feeling a strong sense of ownership over her child. This account emphasizes the need for a plan to regain trust and connection with the grandmother. Many women wanted a clear explanation of the Child Protection system so that they could work towards reunification. It was apparent from group discussion that some women did not understand why their children had been removed and needed clarity about what defined neglect^2^:

Reasons why welfare grabs kids and things and all of that there because they always say neglect and that, you know. (FG 5)

This woman referred to Child Protection as “welfare” and “grabs” as removing her child/ren in a way that left her confused about the expectations of Child Protection.

One woman described how she had battled to regain custody, however, this was not enough for reunification. This woman described feeling like all her attempts never met the expectations of Child Protection. Negative experiences made some women angry, and they blamed the system.

We don’t have a structure; we don’t have a canvas that they expect us to work with – you haven’t given us what you have taken from us so how are we going to work with that? It is like making a cat or a dog chase a piece of a ball, or a ball of string and just go, let’s keep playing with it. (FG 3)

The women acknowledged their need for education starting with a basic understanding of what children need to avoid neglect. There was a general sense of injustice because some women felt unsupported by Child Protection in the decision to remove their child. Women admitted they had not been shown love in their own childhood, identifying the need for practical support. During one focus group, researchers witnessed a woman being asked to leave and were later informed that a decision had been made about her infant being placed in long-term guardianship. Her distress was compounded when authorities discussed issues directly with a social worker, rendering her invisible in the conversation. This incident was discussed with prison guards and was reflected upon and written up in the researcher’s reflective journal.

Women discussed wanting support and information to cope with mothering after release. Some women had commented on their previous release from prison “It was overwhelming, I didn’t know where to start” (FG 4) and “Your role in their life has changed somewhat so navigating that can be quite tricky” (FG 2). For the few that described a positive relationship with their family, the outlook was one of hope. Some women described being grateful their children were well cared for. One woman explained her family’s role in looking after her child,

I couldn’t be happier with the way my son is being brought up at the moment – like it’s very good. He’s doing a better job than probably I could have done to be honest. (FG 1)

Several women who were supported by family had plans for reunification, with a gradual transitioning approach. This emphasizes the importance of family relationships to facilitate support for women after release. Many women reported complex challenges with the caregiver, requiring reconnection and, in some circumstances, with custody as an issue. Caregivers restricting telephone calls or child visiting meant some women did not receive child visits from their children. Some caregivers had become very protective over the children which made it difficult for woman to re-negotiate their mothering role. The need to rebuild trust with her mother was described by one woman,

I don’t necessarily disagree with the way she’s been raising them. It’s just she said I can’t do enough to prove to her that I’m ok now and you know, yep so she worries about that. (FG 1)

This woman discussed how her mother had taken on the mothering role and developed a motherly bond with her grandchildren. The grandmother was seen as the protector of the child and the incarcerated mother did not see this as supporting her to regain custody. This view produced feelings of frustration, anger and helplessness, due to the uncertainty of negotiating the relationship and regaining custody. It was evident some women may need support to improve the relationship and rebuild trust.

Several women described internal conflict and being torn between feeling grateful to the caregiver for caring for their children, leaving the enormous task of parenting, often to their own mother. There were often disagreements with how the caregivers were parenting, particularly in relation to discipline.

I was saying on the phone – ‘no mum don’t do that, ground them’ – you know, ‘don’t smack them these days mum’ – ‘ground them or do this or do’ … don’t always know how they should ummm … . how they should be coping. Yeah, so how do we let them know like, how to work together? – to work together. Yeah, cos me and mum have total different ideas of how to bring children up. (FG 4)

Some women were aware they had not been a good role model and had lost their children’s trust and respect, making discipline challenging. Several women reported they were lenient or did not chastise their children because they felt guilty. Another woman, however, said the opposite,

Cos, I was real strict, I wouldn’t let them do nothing so I need a learn how to mellow it a bit (laugh). Let them have a life, you know (laugh). (FG 4)

Additionally, a few women wanted to learn how to play with their children (various ages) and activities they could do. One woman said:

What to do with them, how to play – yes, I am at a bit of a loss with that, that is a common thing in here. (FG 1)

Some women questioned how to entertain young children and had a lengthy discussion about the influence of technology. Some women believed children of all ages were reliant on technology for entertainment. A few women commented or questioned the harm computer and television screens were causing, as they mentioned it being bad for them, making them “brain dead” or causing behavior that is challenging for parents. It was clear that women had questions to ask about parenting and did not have the opportunity to have questions answered by a trusted professional. In order to reconnect with the children on release, women acknowledged they would need support and education. Some women were aware of waiting times associated with support services and others were not aware of available support. One woman discussed how she had accessed services in the past and yet she said, “there’s nothing”, as her past negative experience with lengthy waiting times had caused her to lose hope. She required immediate support after release,

There’s nothing there really, in the sense for women coming out of prison to help them deal with their kids and what they have dealt with – no one wants their kids to be motherless because they are in jail but at the end of the day what is there, out there for us, to help them – there is nothing. (FG 4)

This negative view of support services led to feelings of isolation and the belief of being unable to break the cycle of incarceration. This woman recognized the immediate support she required was not available and felt unsupported:

So, at the end of the day, you are setting us – they are setting us – women up to come in here. (FG 4)

A few women mentioned the need for a support line (telephone) for mothers and were unaware several services exist. Some women spoke positively about support groups called ‘Second Chances’,^3^ ‘Seeds of Affinity’^4^ and ‘Relationships Australia’^5^ provided for women in South Australia. Many of the women openly engaged in discussion about their parenting challenges, worries and potential ways they could be supported. Women reported they experienced negative emotions and trauma and were seeking connection with children and family.

## Discussion

This current study identified specific needs of incarcerated women regarding their parenting. Four themes were identified, working towards a positive self, communication as a lifeline, supporting and nurturing their children and hopefulness and reconnecting. These themes resonate with other research studies which have identified the needs of women who are incarcerated and attended a parenting program in prison. Common themes include: the effect of separation and incarceration on children, increased knowledge of child development and care, communication, improved child visits, emotional care and child behavior strategies (Eddy et al., 2008; Loper & Tuerk, 2011; Miller et al., 2014; Perry et al., 2009). This study confirmed that women were wanting support with learning skills for writing letters which has been demonstrated as an important form of communication from prison and can reduce parenting stress (Loper & Tuerk, 2006). Interest in learning about practical parenting strategies was also discussed although, many women did not have regular physical contact with their children. The women were seeking clarity about discipline, playing with children, teenagers and some had specific parenting questions. Many women described their children’s behavior deteriorating during their incarceration, experiencing feelings of powerlessness to provide support. This finding confirms earlier reports of powerlessness felt by women during incarceration (Brown & Bloom, 2009).

The women’s accounts of their own childhood experiences indicate that some women were without a positive role model to influence their parenting. A history of abuse is commonly experienced by incarcerated women and has been reported to influence parenting experiences and expectations (Kates et al., 2008). The women needed practical strategies, as they appeared to be overwhelmed by events in their past and current life, with their lives having a vicious cycle of problems which felt almost impossible to escape. Reflection on their own childhood may assist women in gaining an understanding of why some have struggled and can now learn positive parenting approaches. A strength of this exploratory study is the opportunity for incarcerated women to share their views and co-create a parenting education program. However, the findings of this study are based on the views and experiences of women in one prison setting, therefore, may not be generalizable to all women prison populations.

The topic of Child Protection was initiated by the women and revealed emotions such as anger, frustration and confusion. Witnessing the distress of a woman after her baby had been removed, enabled us to see these emotions firsthand. Women wanted to know more about Child Protection and how to reunite with their children. Inclusion of Child Protection as a topic in a parenting education program could be beneficial and this was supported by the requests of the women in the focus groups. This focus is similar to another study which included child custody and legal issues in a prison parenting program and reported positive feedback from women participating (Kennon et al., 2009).

The majority of Aboriginal women in this study expressed a view to have a separate education group. The over-representation of Aboriginal women in Australian prisons makes it imperative to understand their needs and provide education they can relate to (Jones et al., 2018; Sullivan et al., 2019). For this reason, the needs of Aboriginal women will be detailed in another publication. Trauma was apparent in the stories told by the Aboriginal women and the removal of children through Child Protection was particularly evident. They expressed a need to connect with their Elders which concurs with Radke’s view that this way they could build respectful relationships and pass on cultural knowledge (Radke, 2018). Connecting with Aboriginal Elders is an important way to maintain culture and community. These connections have a significant impact on the social and emotional wellbeing of Aboriginal peoples as well as connection with land, culture, spirituality, and family (Dudgeon et al., 2018). As with the non-Aboriginal women, communication and connection was of high importance. Findings suggest that women from non-Caucasian backgrounds may continue to experience racism and discrimination causing additional trauma.

A few women identified negative experiences when attempting to seek support in the community, after release from prison. These experiences appear to have had a negative impact on their motivation to seek further support, thus leading to feelings of isolation as they recognized the enormity of breaking the cycle of incarceration. Nevertheless, other women were aware of several support services available. However, the majority of women did not discuss utilizing available resources and it did not seem to occur to some women that accessing services might be beneficial for their children as previously reported by Brown and Bloom, (Brown & Bloom, 2009). Furthermore, connecting with community services whilst in prison has been identified as improving the uptake of accessing services after release (Sheehan et al., 2011 p. 307).

The current study, while confirming the importance of these parenting needs, also included the need for positive self-care, particularly in relation to the trauma women experienced in their lives. As the women told their stories they revealed trauma experienced, describing domestic violence, difficulties during their childhood, removal of children through Child Protection, separation from their children and family, the threat of deportation, racism and discrimination. The women described many negative emotions experienced as well as mental health conditions reported – for example, depression and anxiety. The experience of trauma has been reported extensively throughout the literature (Abad et al., 2013; Easteal, 2001; Kates et al., 2008; Loper & Tuerk, 2006; Prguda & Burke, 2020; Stone et al., 2015). This finding highlights the importance of considering a trauma-informed approach for parenting education (Easteal et al., 2015; Saxena et al., 2014). The stories shared in the focus groups highlighted the need to create a culture of safety within the group, with the potential to develop peer support and not feel judged. These findings align with what is recommended in the Bangkok Rules and can assist in women feeling safe and avoid-retraumatizing the women (UN, 2011). Parenting education needs to be strength-based and promote healthy connections with children, family, and community (Covington & Bloom, 2007). A trauma informed approach has not been described in previous evaluated parenting programs for incarcerated women.

### Implications and recommendations for a parenting program

Based on the findings from this study a parenting program would need to have short sessions, multiple times a week with up to 8 sessions. The sessions could be presented as individual modules permitting women to continue the program with limited disruption if they miss a session and potentially attend the missed session with another group. A certificate could be awarded to the women after each module and on program completion. Modifications for low literacy levels would need to be incorporated. The program could include topics such as healthy communication with particular attention to writing letters, telephone, and video calls. The program could also include discussion around difficult conversations for example, how to tell children mum is in prison, or how to discuss typical problems for teenagers. Relevant scenarios could be presented for the women to discuss and prepare them for future conversations with their children. A segment about how to plan goals may help the women to think through their problems and the steps required to address them, as well as the supports needed. The women wanted to learn about practical parenting which could include positive discipline, understanding the behaviour of children, labelling emotions, active listening, play and connecting with the caregiver. Therefore, the basic needs of children could be discussed in a practical way to enable women with limited previous positive role modelling to clarify the foundation of positive parenting for the mother and child. Based on the needs of the women information about Child Protection could be beneficial to clarify processes involved and assist women in understanding reunification.

The program would need to align with the current evidence best practice parenting (authoritative parenting style) with the voices of the women at the forefront (Ulferts, 2020). The program would need to convey the understanding that all mothers bring to parenting different ideas, interests, values and cultural influences. Many of the strengths of Aboriginal parenting practices align with authoritative (positive) parenting style which could be highlighted throughout the program and maintain cultural relevance.

Due to the trauma women expressed, it is important that a program is underpinned by a trauma informed approach as per Table 1. Program facilitators need to be educated about a trauma-informed approach and be sensitive to the needs of the women to avoid further trauma. Facilitators would need to demonstrate the belief that recovery is possible and promote learning from each other as a way of building relationships and removing power imbalance. Historical trauma experienced by Aboriginal peoples would be included in the preparation for facilitators and acknowledged in the program. It is recommended that Aboriginal women would be offered a separate group facilitated by an Aboriginal woman. The Australian Law Reform recommend that programs for Aboriginal women must be developed with and delivered by Aboriginal women (ALRC, 2017).

**Table 1:** Six core principles of the trauma-informed approach (Kezelman & Stavropoulos, 2020; SAMHSA, 2014)

Needs and considerations identified by the women	Principles	How program content/foundation could reflect a trauma informed approach
Impact of mental health conditions on learning and engagement Women not wanting to feel judged Trauma and intergenerational trauma	Safety	**Physical and emotional safety** **•** Respectful and engaging communication • Clear explanations • Humane, respectful and empathic and compassionate interactions • Comfortable environment • No judgement from others • Inclusive of all cultures and ethnicity
Parenting expectations outlined Clear information (written, verbal, video)	Trustworthiness	**Clarity, consistency and interpersonal boundaries** • Clear explanation of program content, goals, expectations • Program facilitator should maintain professional boundaries and a friendly disposition • Participation is voluntary and consent gained where the goals, risks and benefits of involvement in the program have been discussed • All agree not to share information outside the program • Provided with simple relevant information
Positive influence of peer support and discussion Shared similar circumstances Learning through others with lived experience	Peer support	**Peer support to encourage recovery and healing** • Provide opportunity for women to find a person to buddy, inside or outside education group • Opportunity for open discussion and exploration • Lived experience person to promote recovery and hope
Address personal aims and individual needs Plan for the future Gain knowledge and understanding from information and open discussion for connection and re-connection with children and family Positive health care strategies Access to parenting information Opportunity to ask questions about parenting Loss of mothering identity	Empowerment, voice and choice	**Maximising choice, control, empowerment, and skill-building** • Program facilitators use a trauma-informed approach to promote recovery and healing • Voluntary program attendance • Choice to book into specific sessions or attend the whole program • Given a choice about participation during education sessions • Opportunity to plan and make goals • Learn skills and build on own strengths • Promote working towards a positive future • Provide an opportunity to discuss motherhood, write to children, learn positive communication • Time to reflect, identify as a mother and ask questions
Feelings of powerlessness were identified Connection with culture and community	Collaboration	**Maximising collaboration and sharing power** • Program founded on needs and views of women • Women seen as the experts of their own experience and minimize the power imbalance • Respectful, de-escalating language to be used to promote effective communication • Discussion will promote sharing of individual stories, culture, values and beliefs • The program will be piloted whereby the women’s views of the program will be taken into consideration and potential changes made • Personal stories shared from others with lived experience
The choice of an Aboriginal only parenting education group Racism and discrimination identified as an issue Trauma of child removal Connect with culture and Aboriginal Elders	Cultural, historical and gender issues	**Responsive to race, ethnicity, culture, and historical trauma** • Encourage learning and sharing about culture and diversity • Encourage women to connect with culture and teach their children about culture • Provide opportunity to hear from Aboriginal Elders • Provide an Aboriginal group for Aboriginal women and a group open to all women, including Aboriginal women (have a choice) • Acknowledge impact of trauma and Australian history, leading to intergenerational trauma • Provide information about Child Protection to optimize support

## Conclusion

The needs voiced by the women, highlighted in this study will assist in the co-creation of a parenting program for women experiencing incarceration in South Australia. Women contributed a substantial amount of rich data to gain valuable insights and explain their complex parenting and relationship challenges experienced whilst being incarcerated. Women reported insufficient opportunities for discussion about their children and lack of appropriate parenting education to meet their needs. Some women did not appear to be aware of community support available or how to instigate access. Women’s main priorities were reconnecting and learning how to support their children through working towards a positive self and improving their communication skills. The need to learn how to improve communication and ways to remain connected were vitally important as was the mother’s role in nurturing and support. The four main themes interpreted from data in this study represent women’s voices reflecting what could be included in parenting education, providing insight into the needs of the woman, child and caregiver. In conclusion the need to learn how to improve communication and ways to remain connected were vitally important as was the mother’s role in nurturing and support. The themes provide a starting point for the development of a parenting education program to address the challenges and concerns of women experiencing incarceration. There is scope to further investigate the influence of culture on parenting practices and pilot and evaluate a program with a focus on the cultural needs and relevance for participants.

## Data Availability

Identifiable material was removed from the data collected however, it is possible that indirectly identifiable information could be gathered from the raw data and therefore data sets will not be shared.

## Footnotes

1 Hereafter referred to as Aboriginal, the authors acknowledge the diversity within Aboriginal cultures.

2 Neglect is defined as the failure to provide a child, basic needs for example, food, shelter, clothing, supervision and medical care (NAPCAN prevent child abuse and neglect 2022).

3 Second Chances SA is a non-profit volunteer organisation supporting people in prison, their children and families with programs.

4 Seeds of Affinity is a non-profit volunteer organisation established by and for women who have experienced incarceration

5 Relationships Australia is a non-profit, charitable organisation providing health and wellbeing services to vulnerable and disadvantaged people.
